# Determinants of Choice and Wine Consumption Behaviour: A Comparative Analysis between Two Counties of Romania

**DOI:** 10.3390/foods11081110

**Published:** 2022-04-13

**Authors:** Anca Monica Brata, Daniel I. Chiciudean, Vlad Dumitru Brata, Dorin Popa, Gabriela O. Chiciudean, Iulia C. Muresan

**Affiliations:** 1 Faculty of Environmental Protection, University of Oradea, 410048 Oradea, Romania; abrata@uoradea.ro (A.M.B.); dopopa@uoradea.ro (D.P.); 2 Department of Economic Sciences, University of Agricultural Sciences and Veterinary Medicine, 400372 Cluj-Napoca, Romania; daniel.chiciudean@usamvcluj.ro (D.I.C.); iulia.muresan@usamvcluj.ro (I.C.M.); 3 Faculty of Medicine, “Iuliu Hatieganu” University of Medicine and Pharmacy, 400000 Cluj-Napoca, Romania; brata_vlad@yahoo.com

**Keywords:** wine consumers’ behaviour, principal component analysis, market orientation, purchase

## Abstract

Wine, one of the world’s oldest and most popular beverages, has a distinct variety matching a diverse base of consumers worldwide. The study was conducted in two counties of Romania in order to identify consumers’ perception towards wine consumption, as well as the driving factors behind wine consumption and the decision process of choosing a certain type of wine. Thus, four factors were identified through principal component analysis: intrinsic cues and consumers’ experience, extrinsic cues and origin, notoriety and the label and package of the wine, correlating them with the socio-demographic characteristics of our respondents. It might be concluded that the intrinsic cues and consumer experience ranked highest among the priorities of the participants between 35 and 45 years old when choosing a certain type of wine. Additionally, notoriety was more valued by people with higher income, and people with experience in the domain inclined to pay more for a bottle of wine.

## 1. Introduction

Wine is a popular and long-established alcoholic beverage that has been consumed for hundreds of years [1], with moderate consumption being associated with significant health benefits [1,2,3,4,5], whereas excessive alcohol consumption is considered a major risk factor for various diseases [6,7,8]. Nonetheless, data regarding the recommended dose for a healthy life remain equivocal [9], making the product somehow controversial [10]. Numerous research undertaken on this topic [11], the elements influencing wine purchasing being explored by many experts, demonstrate its growing popularity as a product category, as well as in its consumption behaviour [5,6,7,8,9,10,11,12,13,14,15]. The most frequently identified factors refer to the wines’ geographical origin [10,12,13,14,16], the content of alcohol [10,17] or its notoriety, reflected in the number of distinctions received [10], but also the type of grapes, year and origin of harvest [17]. Moreover, it has been previously observed that the individuals’ socio-demographic characteristics often influence the type of wine purchased and consumed with regards to age, gender and mostly income [18,19,20,21,22]. In Romania, wine studies are primarily concerned with consumer behaviour and information sources at the time of purchase [12,13,14], not taking into consideration the factors influencing the consumers’ decision to purchase. Furthermore, the Romanian research has mainly focused either on the intrinsic attributes (sensory characteristics) [23] or the extrinsic ones (brand, notoriety, price, packaging, etc.) [12,23], which is similar to the findings of Mueller et al. in Australia, without considering them as a whole when referring to the complex product that is wine [24].

Aside from the importance for academics, the determinants of impact at the point of purchase are equally crucial for wine producers who must develop new market tactics to promote consumption [15] and consequently adjust their supply according to the consumers’ preferences [18,19,20,21]. Among the European countries, Romania ranks seventh, after Russia, in terms of wine production, with 3.6 million hectolitres in 2020 [22]. The wine production improved in terms of both quality and quantity [25], with 40 new wine producers having just recently entered the wine market [25]. Nevertheless, when it comes to wine consumption, the situation is very different; in 2020, the amount of wine consumed per capita was 21.1 L, down from 23.4 L in 2019 [26], confirming that Romanian consumers do not have a wine culture such as other European countries such as France [27], Italy [28], Spain [29] or Portugal [30]. The most consumed alcoholic beverage in Romania remains beer with 87.8 L per capita in 2020 [26].

The present study aimed to determine the consumers’ perception towards wine consumption by identifying the factors influencing the consumption, for better understanding the characteristics of the wine consumers and the attributes that they are seeking when deciding to purchase wine, as well as to help the Romanian stakeholders involved in the wine area of production and commercialization to improve their marketing strategies according to the market’s needs and contribute to an increase in domestic consumption.

## 2. Literature Review

Wine has been regarded as one of the oldest products created by humans and further transformed into the popular beverage of today [1]. It is nowadays considered a combination of art and science, integrating elements such as creativity and technology [31] in order to provide the variety matching its diverse base of consumers worldwide [32]. Research has recently showed a promising direction in the field of emotions sparked by wine consumption, Ferrarini et al. developing a list of 16 Italian adjectives that could describe various emotions triggered by wine tasting [33].

From a cultural standpoint, wine is traditionally connected with Southern Europe, while beer is associated with Northern Europe [34]. Silva et al., analysed consumers’ behaviour in two different European countries in terms of preferred type of purchased alcohol—Portugal and the Netherlands [34]. Thus, there is a significant distinction between the quantity of wine and beer consumed in both countries, assessing the motivations and factors influencing the decision of choosing one of the two. Although wine consumption in Portugal has decreased over the years, with beer growing more popular, it still remains the most consumed alcoholic beverage in the country [34]. In both Portugal and the Netherlands, wine is more likely consumed in domestic settings, whether at home or over at family or friends, with Portuguese consumers also choosing wine more frequently in restaurants, when compared to their Dutch correspondents [34]. Additionally, wine is regarded as complementary to certain foods and is strongly associated with special occasions, while beer is viewed as a more “informal” beverage. Differences between the consumption pattern in Southern and Northern European countries also occur due to the importance that certain populations provide to various meals of the day. To this matter, Portuguese were more likely to consume wine at lunch, dinner and at the weekends, while, for the Dutch, alcohol intake during the weekdays and the quantity of alcohol consumed with meals were significantly lower, most of them resorting to drinking during the weekends [34].

Nevertheless, choosing and purchasing a certain type of wine is more thorough, with many factors depending not only on the product, but on the consumer as well. Studies conducted in this domain have managed to create several consumer profiles and identify the factors they mostly rely on when purchasing wine. The so-called “extrinsic characteristics” of the wine consist of the first things consumers come into contact with when purchasing a wine: price, label, prizes and awards, volume, country of origin, alcohol percentage and various store promotions [35]. However, consumers with a developed wine culture and high incomes are more likely to rely on the intrinsic cues of the wine, such as taste, colour, flavours, smell and the varieties of grapes used, [36,37,38,39]. This component is also strongly associated with previous experiences, whether the customer has tasted that certain type of wine before, recommendations from other consumers, or simply the customer’s psychological association of an event with that said wine [10,40,41,42,43]. The amount to which each one of these variables influences the process of wine purchasing depends on many factors, such as the customer’s country and wine tradition, the involvement of the consumers, income, education level and, of course, personal preferences. However, traditional wine consuming countries differ from newly emerging countries in the field of wine culture, with Western and Southern Europeans mostly relying on the intrinsic characteristics of the wine [36], while consumers from China and Russia are more interested in extrinsic cues: the medals earned, price, country of origin, landscape certification, etc. [16].

Ferreira et al., concluded that, for a proper evaluation of wine quality, consumers need knowledge and experience [35]. Thus, based on these dimensions, two types of consumers emerged: least knowledgeable and very knowledgeable [35]. The first segment evaluates the wine quality based on attributes such as brand, food pairing, alcohol content and wine image, while the second segment appreciates its quality based on region, grape variety and alcohol content. Italian consumers were grouped into four categories based on their characteristics and purchasing behaviour highlighting the importance of brand in the case of wine [35]. The “loyal group” comprises young consumers with a higher income, the “habitual group” consumes wine less frequently than all the other groups. The “variety seekers” are generally old and have high incomes while the “switchers” represent the largest group rather seeking diversity instead of a certain brand [35,36]. Wine is, indeed, a special product that is able even to improve one’s social image, so it has been stated that narcissistic people consume higher amounts of wine due to its social attractiveness compared to individuals with lower degree of narcissism [37].

Moreover, when it comes to wine industry and wine consumption, there are certain factors that need to be addressed by the producers regarding the type of consumer of their wines. Chaney analysed the decision making of UK consumers when it comes to wine purchase, revealing that little to no proper research by the customer is conducted prior to the purchase, the consumers reflecting more on the options they were to find in the store [44]. Further extensive research proved that, indeed, the aspect and attractiveness of the label plays a more important role in the purchase and consumption of wine [45,46,47,48,49]. Consumers are also more likely to choose a wine whose name has a certain degree of notoriety [42,49] or a wine which they have previously tried [10] or has been recommended by acquaintances [42,43]. Factors such as the geographical origin of the wine and type of grapes also play an important part when deciding over the type of wine one is going to pick out of a multitude of choices [42,50,51,52]. Studies have also shown that people that read about and are generally interested in wine culture tend to check for additional information when making the decision of choosing a certain type of wine, either reading about it at home or in the store [42,53]. Additionally, studies performed by Jaeger et al. in New Zealand and by Bărbulescu in Romania revealed that participants tended to assess the extent to which the wine they chose matched the food they were about to prepare or eat in restaurants, looking for the meal and wine to go well together [42,54]. Other factors and information people taking part in the study also considered when choosing their wine were the number of competitions or distinctions the said wine had previously received or whether the considered wine was on a promotional display in the store [42]. The average alcohol content of beverages in general and wine in particular is also frequently taken into account by consumers [42,55].

The amount of influence the previously mentioned factors have upon the consumer’s process of decision making is also strongly correlated to certain socio-demographic aspects. Age and gender play an important role and firmly influence wine taste of consumers [41,46]. A study by Barber et al. that analysed participants from the state of Connecticut revealed that females and people in the 30–40 years age group tend to analyse more thoroughly the aspects leading to the decision of purchasing a certain type of wine [39]. Another study performed by Saad in 2005 on participants from the USA also concluded that nearly half of the adult female population preferred wine over other alcoholic beverages [56].

Another important factor that considerably impacts the decision and choice of customers when it comes to wine is represented by the income they have [39,40,41]. Blaylock and Blisard analysed the wine consumption of US men, indicating that men tend to consume more wine if they are under 65 years of age, have a steady and high income and are also highly educated [40]. Moreover, the study revealed that male heavy wine drinkers were over 65 years of age, but also exercised regularly and had higher incomes [40]. Gunay and Baker analysed the preferences of Turkish wine consumers, establishing a correlation between income and education levels and factors influencing their decision of wine purchase, with higher education and income individuals orienting themselves towards quality superior wines, while people with lower incomes and education were more attracted by the low price of the product or various promotional displays [56].

Additionally, the extent to which consumers are interested in the wine industry and culture also influences the way they tend to choose and select certain types of wines and the quantity they buy [42,43,56]. Hussain et al. conducted a study with the aim of identifying the determinants of wine consumption of US customers [41]. The study identified significant correlations between the amount of wine consumed and the involvement of the consumers in wine industry and culture [41]. However, when it comes to the thought process and decision making regarding which wine to choose, research has shown an important distinction between consumers with different levels of involvement [56,57]. Barber et al. analysed the so-called method of purchasing a certain type of wine of participants from the state of Connecticut with different levels of involvement, revealing that consumers with lower involvement inclined more on external features of the wines, such as the front label, which provided information about the country of origin of the wine, type of grapes, or whether it was a vintage type [46]. Moreover, it was also revealed that novice enjoyers of wine were more price sensitive in comparison to people more involved with wine [46]. Thus, studies analysing consumers’ behaviour in relationship to wine consumption have concluded that people with an important wine culture and involvement are less likely to be influenced by the extrinsic cues of the product, relying more on the intrinsic characteristics of the wine [41,46,56].

Ferreira et al. consider that, for a proper evaluation of wine quality, consumers need knowledge and experience [35]. Thus, based on these dimensions, two types of consumers emerged: least knowledgeable and very knowledgeable [35]. The first segment evaluates the wine quality based on attributes such as brand, food pairing, alcohol content and wine image, while the second segment appreciates its quality based on region, grape variety and alcohol content. Italian consumers were grouped into four categories based on their characteristics and purchasing behaviour highlighting the importance of brand in the case of wine [35]. The “loyal group” comprises young consumers with a higher income, the “habitual group” consumes wine less frequently than all the other groups. The “variety seekers” are generally old and have high incomes, while the “switchers” represent the largest group, seeking rather diversity instead of a certain brand [35,36]. Wine is, indeed, a special product that is able even to improve one’s social image, so it has been stated that narcissistic people consume higher amounts of wine due to its social attractiveness compared to individuals with lower degree of narcissism [37].

Nonetheless, one of the most important aspects influencing both the wine consumer and producer is the country and region in which certain wine is marketed [39,40,41,42,43,57,58,59]. Research has shown that the previously mentioned factors are crucial and valid regardless of the location. However, certain differences and aspects are to be seen in correlation to specific areas and regions, considering the distinctive features of cultures around the globe. Another attribute that could increase the consumers’ preference for one type of wine or the other is sustainability, being reflected either in the production methods or in its local origin [60]. Gunay and Baker analysed the behaviour of Turkish wine consumers, revealing that red wines and wines with a higher alcohol content are preferred in this type of market, due to the other local beverages with a high content of alcohol [56]. St James and Christodoulidou conducted research on factors influencing wine consumption in South California, showing that many participants named the health benefits of wine as a reason for consuming wine [59]. With research being continuously improved and conducted in this field of interest, it has been revealed that moderate wine consumption can have a beneficial effect, helping reduce oxidative stress in the cardiocirculatory system and prevent a multitude of chronic diseases [58,59]. This could also influence people into settling for wine, to the detriment of other alcohol beverages.

When it comes to wine consumption in Romania, a study by Ladaru and Beciu revealed that most Romanian consumers participating in the study bought their wine from specialized stores, due to more information being available in this type of location [12]. Moreover, the study showed that the type of grapes, year and origin of harvest play an important role in the process of purchasing a certain type of wine [12]. Nevertheless, Bărbulescu analysed both the Romanian producers marketing their wines through restaurants and consumers buying a certain type of wine in the restaurants, concluding that the Romanian consumer tends to check multiple information sources online, before choosing a specific type of producer and wine [54].

## 3. Materials and Methods

The main objective of the research was to identify the consumers’ perception towards wine consumption. At the same time, it was considered necessary to identify the factors affecting the consumers’ wine consumption.

### 3.1. Research Methodology and Questionnaire Design

The research consisted of two main phases: (i) the first phase represented by the literature review in order to determine the factors influencing wine consumption and wine consumers behaviour trends; (ii) the second phase consisted of a survey among wine consumers from Cluj and Bihor counties of Romania. The survey was based on a questionnaire developed on previous research [61,62,63]. The first part of the questionnaire allowed the researchers to collect data about the socio-demographic characteristics of the respondents, while the second part consisted of a set of 21 items, evaluated on a Likert-type scale from 1 to 5, where 1 means not important at all, while 5 means very important. At the same time, in this part, two questions related to price and consumption frequency were addressed as well. A pilot test on 10 respondents was conducted in order to check the reliability of the research instrument.

### 3.2. Sample Size and Data Collection

The study was based on a convenience sample of 285 wine consumers from Cluj and Bihor counties. The survey was based on a self-administrated survey during which each participant was informed about the aim of the survey and the processing of their personal data according to the principle laid down in the General Data Protection Regulation of the European Union. A total number of 350 questionnaires were distributed, and 285 were validated in the end.

Out of the total number of respondents, 45.3% were female and 54.7% were male, providing a proper gender balance for the study. A total of 23.5% of the participants graduated high school, while 46% had a university degree and 30.5% a postgraduate degree. Regarding the age distribution, most of the respondents were over 35 years old, with almost a third (32.6%) being over 45 years old. The proportion of respondents belonging to the 18–25 and 25–35 years groups were similar (19.6% and 19.3%, respectively). When it comes to the place of residence, the participants were fairly distributed, with 49.5% reporting their place of residence in Bihor County, while 50.5% resided in Cluj County. Regarding the respondents’ income, most of them earned more than RON 2000 monthly, with 37.2% of them reporting salaries greater than RON 3000. On the other hand, 12.6% of the participants were making less than RON 1000 per month, and 22.5% earned between RON 1000 and 2000 monthly (Table 1).

### 3.3. Data Analysis

The data were analysed using SPSS 26.0 software package (SPSS Inc., Chicago, IL, USA). The descriptive statistics was used to analyse the socio-demographic profile and the consumption frequency. The principal component analysis was used to reduce the dimensionality of the 21 items that evaluate the attributes that influence their wine consumption behaviour. The retained factors had an eigenvalue higher than 1. Cronbach’s alpha coefficient was calculated to test the internal consistency of the items. The value obtained was above 0.6, indicating a good internal consistency of the date. Furthermore, the Mann–Whitney U and Kruskal–Wallis tests were used to assess any significant differences among the importance of the factors towards wine consumption behaviour and socio-demographic characteristics.

Principal factor analysis was conducted in order to assess the dimensionality of the 21 items. Barlett’s test of sphericity was significant (Chi-Square = 3384.530; *p* < 000) and the Kaiser–Meyer–Olkin (KMO) measure of sampling was 0.867, indicating that the considered data were adequate for principal component analysis (PCA). Values of 0.6 or above for the KMO measurement indicates adequate data for PCA [64,65]. The PCA with varimax rotation of the 21 variables resulted in a four-component solution explaining 62.481% of the total variance. Factors with eigenvalues greater than one were selected. We used Cronbach’s alpha reliability coefficient in order to evaluate the internal consistency of each component, obtaining an overall value of 0.915. An acceptable reliability coefficient higher than 0.6 should be considered [66].

## 4. Results

### 4.1. Consumer Perception on Main Factors Influencing Wine Consumption

The four components resulted from the PCA, each with their corresponding variables were detailed in Table 2. The first component analysed intrinsic cues and consumers’ experience regarding wine consumption and explained 37.774% of the total variance, with a reliability coefficient of 0.897. It consisted of eight variables and had a mean of 3.88 and a SD of 0.379. Wine taste, flavour, smell and clarity were the most significant factors taken into account by respondents, while alcohol content was considered the least important aspect belonging to this component, with a mean of 3.2 and SD of 1.24. The respondents’ previous experiences with certain wines also had a significant impact on the decision of purchasing a certain type of wine, with a mean of 3.95 and SD of 1.198.

The second component was correlated with extrinsic cues and origin of the wine, explaining 10.841% of the total variance, with a reliability coefficient of 0.805, and a mean of 2.82 ± 0.539. It consisted of six variables, with respondents ranking the quality/price ratio (3.59 ± 1.328) higher than the low price of wine (2.55 ± 1.276) or existing promotions when purchasing a certain type of wine (2.49 ± 1.195). Even though the country of origin was an important factor taken into account by consumers (2.99 ± 1.213), respondents were more likely to choose a wine made in Romania (3.29 ± 1.310). Out of the variables belonging to the second component, the bottle volume was ranked as the least important one, with a mean of 1.98 ± 1.185.

The “Notoriety” component assessed the importance of five variables (vineyard, ecological certificate, awards, wine region and brand), representing 7.589% of the total variance, with a reliability coefficient of 0.813 and a mean of 3.1 ± 0.301. Vineyard represented the most significant factor taken into consideration by respondents (3.54 ± 1.142), followed by wine region (3.39 ± 1.147) and brand (3.2 ± 1.14), while the awards that a certain wine has received were perceived less important by participants (2.83 ± 1.273).

When it comes to label attractiveness and packing, these variables were comprised in the “Label and package” component, representing 6.278% of the total variance, with a reliability coefficient of 0.882, and a mean of 2.86 ± 0.0071. The package ranked higher than the attractiveness of the label, with a mean of 2.92 ± 1.218, comparing to a mean of 2.82 ± 1.173.

### 4.2. Socio-Demographical Factors Affecting the Consumer Behaviour of Wine

The relationship between the socio-demographic characteristics of the respondents and the four components resulted from the PCA were illustrated in Table 3. When it comes to gender differences regarding the decision process of choosing a certain type of wine, the only distinction was reported in the “Notoriety” component (*p* = 0.029), where men (3.33 ± 0.693) were more likely to take into consideration a more renown wine in comparison to women (2.98 ± 1.084).

Consumers’ behaviour also diverged depending on the age distribution, with people between 35 and 45 years old being the ones relying the most on the intrinsic cues of the wine and their previous experience (4.1 ± 0.799; *p* < 0.001). On the other hand, people from the youngest category (18–25 years old) took more into consideration the extrinsic cues and the origin of the wine (3.09 ± 0.685; *p* < 0.001). This fact could be closely correlated to the education level of the respondents, where participants who have only graduated high school also relied most on the extrinsic cues and the origin of the wine (3.126 ± 0.769; *p* < 0.001), potentially due to a lower number of people obtaining their university or postgraduate degree before the age of 25. When it comes to the notoriety or the label and package of the wine, no statistical differences were noted between the age groups (*p* > 0.05).

Additionally, wine consumption is strongly influenced by the consumers’ monthly income, our analysis depicting significant differences across all four components of PCA. People with higher incomes (over RON 3000 monthly) were more likely to choose a wine based on its notoriety, compared to participants from different income categories (3.38 ± 0.768; *p* < 0.05). People with incomes between RON 1000 and 2000 paid more attention to the label and package of the wine (3.16 ± 1.011; *p* < 0.05), while participants reporting incomes lower than RON 1000 per month made their decision by considering both the intrinsic cues of the wine and their previous experience (4.11 ± 0.525; *p* < 0.01) and extrinsic cues and wine’s origin (3.08 ± 0.728; *p* < 0.05).

When it comes to the price per bottle, participants were more likely to consider the intrinsic cues and their previous experiences as the most important factors for a type of wine costing more than RON 100 (4.12 ± 0.577; *p* < 0.001). Moreover, for the same price range of the wine bottle, notoriety was also a significant element considered by consumers (3.42 ± 0.576; *p* < 0.05).

Regarding the two counties in which the study was conducted, we reported no significant differences between respondents regarding the intrinsic cues of the wine, their previous experiences with it or the notoriety of the said wine. Nevertheless, participants from Bihor county were more likely to rely on the extrinsic cues and origin (2.80 ± 0.981; *p* < 0.05), while people from Cluj county focused more on the label and package than their neighbours (3.05 ± 1.048; *p* < 0.05).

Our analysis also assessed the relationship between consumption frequency and the four components of the PCA, concluding that differences between participants occurred regarding the intrinsic cues of the wine and their previous experiences, with respondents drinking wine once a month being the ones relying on this component more than other respondents (4.04 ± 0.432; *p* < 0.05).

## 5. Discussion

The results of the study, which aimed to determine the most important factors influencing wine consumption even during the decision-making process, highlight the fact that the Romanian consumers appreciate this type of alcoholic beverage mostly for its intrinsic cues, with “taste” being the most important, confirming previous studies [67,68,69]. In general, wine sampling is limited in stores [44], even if its impact on repurchase is consistent [70,71]. Even though taste is the most significant quality of evaluation, most wine purchases do not come with the opportunity to test the product before the purchase [72,73]. Moreover, tasting before purchasing a certain type of wine is considered important even if it refers to the same brand, provided the fact that taste could be different from one year to another [74]. Given the importance of taste within the process of wine’s purchase, producers and wine-sellers have the option to increase the sampling at the place of vending as a strategy to stimulate the purchase [72]. Previous findings show that most Romanian consumers bought their wine from specialized stores, due to more information being available in this type of location so the testing campaigns should be frequent in these locations [12]. Aside from taste, flavour, scent, and clarity are other significant quality characteristics for Romanian customers. Because clarity is the first quality feature that an expert inspects [75], producers should consider it when introducing new items on the market, with consumers also attentive to this quality attribute [76]. The alcohol content was evaluated as the least important intrinsic attribute, reinforcing previous studies [77,78,79,80,81]. In many circumstances, the consumer’s buying decision is influenced by previous experiences, such as a sense of loyalty to a brand or a happy memory linked with a specific variety of wine, due to the impossibility of tasting different wines at the moment of the purchase. Previous beverage or food experiences are undoubtedly significant for present consumption, according to the theory of evaluative conditioning, which claims that in the instance of wine, previous consumption will affect present choice for certain types of wine [82].

Apart from intrinsic cues, extrinsic characteristics are also key variables in influencing customer’s perceptions of wine, assisting the consumer in making a purchase decision, especially in the absence of tasting [83]. Additionally, as compared to other commercial items, wine has a lot more extrinsic cues that can influence a consumer’s perception and purchase choice. Extrinsic cues were the second most important component of influence for Romanian consumers; the respondents selecting the quality/price ratio as the most important factor of influence on wine, out of the six comprising factors, ranking it higher than the variable “low price of wine.” Price is also considered an important driving factor in the decision of purchasing and repurchasing, as well as liking a certain type of wine or desiring to taste one [84,85]. The frequent association between wines with low price-low quality and high price-high quality underlines the fact that the price/quality ratio is more important for consumers than the price itself [86,87]. Boncinelli et al. also observed the decline of price importance for consumers when choosing a certain type of wine [88]. The other variables such as “high bottle volume” or “existing promotions” are considered less important for the Romanian consumer and are deriving from the previously mentioned phenomenon. The variable “high bottle volume” is the least important variable from all the variables analysed. A cross sectional study revealed that consumers tend to associate the weight of the bottle to the quality and the price of the wine [89]: however, in the case of Romanian consumers, purchasing a higher quantity does not seem that important.

The country of origin was an important factor taken into account by Romanian wine consumers, although not as important as in other countries [90,91,92]. Chinese customers, for example, are strongly influenced by the country of origin [93]. When it comes to the Romanian respondents, a wine that is made in Romania will create a better impression; a fact explained by the relatively low quantity of imported wine (443.3 hL in 2020) on the Romanian market compared to the domestic utilisation (4294.8 hL) [26]. The results are very important for the Romanian producers, since the consumers prefer domestic wines, with wine sellers having to adjust their offerings to the demand for Romanian wines, instead of foreign ones.

Wine’s notoriety is also important for the Romanian consumers, the vineyard being the most significant factor taken into consideration by respondents out of the analysed variables in this category, followed by wine region and brand, further confirming the results obtained by previous Romanian research, which stated that the type of grapes, year and origin of harvest play an important role in the process of purchasing a certain type of wine [12]. The interesting fact is that the awards that a certain wine has received were perceived less important by participants. It was observed that the display of awards on wines is associated with scepticism among the consumers, even though it remains an effective marketing tool [94]. Herbst and Von Arnim assessed the importance of wine awards on the South African consumer, revealing that the high number of competitions has reduced the value of the awards [93]. The issue of wine awards is very complicated from the consumers’ perspective since there were observed to be two types of consumers: low-involvement consumers willing to pay a higher price for an awarded wine, and high involvement consumers, distrusting awards and being negatively influenced by this aspect [94]. Another study concluded by Monteiro et al. revealed that the consumers’ purchase intentions are positively influenced by the awarded bottles mainly for those wines that are meant to be consumed within a social environment [95].

For the Romanian consumer, vineyard is more important than brand due to a large number of famous vineyards across the country. Even if unfamiliar with the brand, consumers tend to take into account the vineyard origin more, in comparison to the brand. Nevertheless, it has been observed that, mainly in social media, brand is indeed less important [96].

The label attractiveness and packing are not very important for the Romanian consumers, unlike other studies indicating their impact in shaping the consumers preferences for certain types of wine [97]. It has been observed by Barrena et al. that the brand loses importance when it comes to label innovations such as thermo-sensitive and aroma labels for wine [38]. Thus, Romanian producers should rethink their label strategy and try to attract the Romanian consumers who are used to more typical labels, for obtaining a better share of the market.

The results indicate that there is a weak relationship between the socio-demographic characteristics of the respondents and the four components resulted from the PCA, with notable distinctions being found in the “Notoriety” component, confirming the results obtained by Forbes [98], according to which gender is not a useful variable for understanding the wine market. It appears that men tend to care more about the wine’s fame; a fact that could be explained by their better knowledge of the Romanian wine’s market than in the case of women.

The socio-demographic variable that strongly influences wine consumption is the consumers’ monthly income; the results showing that individuals with higher income are more likely to purchase a more notorious wine, while the individuals with medium incomes take into consideration, and rely more on, the label and package of the wine. The present results offered valuable information for the wine producers and sellers referring to the factors influencing the consumers’ decision of purchase, considering that previous research dealt with the consumer behaviour and information sources at the time of purchase [12,13,14].

## 6. Conclusions

This study analysed the factors affecting the Romanian consumer of wine within the context of a relatively low consumption per capita, despite Romania being among the most important ten European wine producers. Identifying the factors influencing consumption could help the producers improve their marketing strategies and obtain a better competitive advantage on the Romanian market.

The results show that the main factors affecting wine consumption among the investigated population are represented by intrinsic cues and consumer experience, extrinsic cues and origin, notoriety and label and package. The intrinsic cues and consumer experience ranked highest for both male and female between 35 and 45 years old. Among the intrinsic factors, taste is one of the most important with direct influence on the repurchase of the product. Still, the number of producers or retailers who offer wine tasting or samplings within stores is very limited; therefore, the consumer faces the situation of uncertainty related to the product and may perceive the purchase as having a certain degree of risk. By adopting the sampling strategy or the tasting campaigns, both retailers and producers could benefit from increased purchases. It has also been observed that the consumer’s buying decision is influenced by previous experiences (loyalty to a brand, or happy memory linked with a specific variety of wine) so the producers or the retailers should increase their marketing strategies in order to build strong brands so that the consumers could easily associate the brands’ name to a specific moment in their lives. Clarity is the first intrinsic attribute that is evaluated by a wine expert, so the actors involved in wine production and commercialization should take into account this specific feature whenever a new variety of wine is launched on the market.

The extrinsic cues and origin characteristics are more important for younger consumers with lower income. The notoriety is most appreciated by consumers with higher monthly incomes. It was noticed that the consumers are willing to pay more for a bottle of wine as their experience in the domain increases. It has been noticed that for Romanian consumers wine labels are not important at the purchase moment, but innovative labels could represent a changing element in building new marketing strategies and obtaining a better advantage in the competitive market.

The results reveal important aspects related to wine consumption in Romania, offering valuable information for future marketing campaigns, taking into consideration the new trends related to on-line marketing.

The main contributions of this paper refer to valuable and useful information for the stakeholders involved in the process of wine production and selling, by offering important data that could help them build the proper marketing strategies that could help increase the sales volume, while creating a cultural identity for Romanian wine, which, until now, has failed to develop.

Limitations are linked to the convenient sample used during research, limited to two counties of Romania and the socio-demographic characteristics of the research area, which may influence the final results of the study and might be different from those that could be recorded nationally. Future research will approach all the counties from the North-West region of Romania in order to establish the main behavioural patterns of Romanian wine consumers and to identify the factors that affect their wine consumption, in order to offer proper information to the stakeholders involved in wine production and commercialization so that it will become the basis needed for building proper marketing strategies and to increase internal wine consumption, and, even more, to build a cultural identity for Romanian wine following other European countries.

## Figures and Tables

**Table 1:** Socio-Demographic Profile of the Respondents.

Characteristics (n = 285)	Variables	%
Gender	Female	45.3
	Male	54.7
Education	High school	23.5
	University degree	46
	Postgraduate degree	30.5
Age	18–25 years	19.6
	25–35 years	19.3
	35–45 years	28.4
	>45 years	32.6
Residence	Bihor County	49.5
	Cluj County	50.5
Monthly income	RON < 1000	12.6
	RON 1000–2000	22.5
	RON 2000–3000	27.7
	RON > 3000	37.2

**Table 2:** Principal component analysis on wine consumption.

Eigenvalue	Variance %	Factor	Item	Factor Loading	Mean	SD
7.932	37.774	Intrinsic cues and consumers’ experience α = 0.897 mean = 3.88 ± 0.379	Smell	0.816	4.06	1.033
			Clarity	0.780	3.98	1.107
			Taste	0.776	4.45	1.001
			Colour	0.766	3.78	1.140
			Variety/Varieties used	0.668	3.56	1.210
			Flavour	0.663	4.12	1.121
			Alcohol content	0.649	3.20	1.240
			Previous experiences	0.539	3.95	1.198
2.277	10.841	Extrinsic cues and origin α = 0.805 mean = 2.82 ± 0.539	High bottle volume	0.792	1.98	1.185
			Low price	0.787	2.55	1.276
			Promotions	0.713	2.49	1.195
			Price/Quality ratio	0.589	3.59	1.328
			Country of origin	0.505	2.99	1.213
			Made in Romania	0.479	3.29	1.310
1.594	7.589	Notoriety α = 0.813 mean = 3.17 ± 0.301	Vineyard	0.726	3.54	1.142
			Ecological certificate	0.698	2.92	1.269
			Awards	0.669	2.83	1.273
			Wine region	0.586	3.39	1.147
			Brand	0.585	3.20	1.140
1.318	6.278	Label and package α = 0.882 mean = 2.86 ± 0.071	Label attractiveness	0.871	2.82	1.173
			Packing	0.853	2.92	1.218
Total variance %	62.481, α = 0.915					

**Table 3:** Relationship between the socio-demographic characteristics and PCA-resulted components.

Characteristics	Variables	Intrinsic Cues and Consumers’ Experience	Extrinsic Cues and Origin	Notoriety	Label and Package
Gender	Male	4.04 (0.509)	2.82 (0.749)	3.33 (0.693)	2.85 (1.037)
	Female	3.73 (1.132)	2.61 (1.049)	2.98 (1.084)	2.88 (1.236)
*p*-value		0.178	0.240	0.029 *	0.754
Age	18–25	3.97 (0.588)	3.09 (0.685)	3.26 (0.600)	2.845 (0.775)
	25–35	3.47 (0.1248)	2.22 (1.097)	2.75 (1.168)	3.00 (1.362)
	35–45	4.10 (0.799)	2.69 (0.772)	3.25 (0.907)	2.80 (1.182)
	>45	3.96 (0.644)	2.86 (0.847)	3.31 (0.795)	2.84 (1.109)
*p*-value		0.000 ***	0.000 ***	0.120	0.393
Education	High school	3.99 (0.530)	3.126 (0.769)	3.205 (0.647)	3.05 (0.893)
	University degree	3.96 (0.608)	2.73 (0.777)	3.22 (0.785)	2.79 (1.103)
	Postgraduate degree	3.74 (1.281)	2.42 (1.047)	3.07 (1.204)	2.82 (1.313)
*p*-value		0.806	0.000 ***	0.899	0.451
Monthly income per person	RON < 1000	4.11 (0.525)	3.08 (0.728)	3.22 (0.63)	3.04 (0.859)
	RON 1000–2000	3.74 (0.846)	2.71 (0.962)	3.03 (0.937)	3.16 (1.011)
	RON 2000–3000	3.69 (1.135)	2.53 (1.003)	2.98 (1.091)	2.69 (1.225)
	RON > 3000	4.08 (0.655)	2.76 (0.806)	3.38 (0.768)	2.75 (1.17)
*p*-value		0.003 **	0.048 *	0.018 *	0.05 *
County	Bihor	3.80 (1.081)	2.80 (0.981)	3.17 (1.041)	2.68 (1.181)
	Cluj	4.00 (0.558)	2.65 (0.813)	3.16 (0.75)	3.05 (1.048)
*p*-value		0.858	0.020 *	0.124	0.023 *
Price/750 mL bottle	RON < 12	4.01 (0.414)	3.47 (0.711)	3.26 (0.684)	3.53 (0.595)
	RON 12–50	3.86 (0.772)	2.93 (0.807)	3.22 (0.851)	2.75 (1.060)
	RON 50–100	3.9 (1.107)	2.31 (0.917)	2.99 (1.060)	2.94 (1.107)
	RON > 100	4.12 (0.577)	2.21 (0.801)	3.42 (0.576)	3.05 (0.863)
*p*-value		0.000 ***	0.251	0.037 *	0.104
Consumption frequency	Daily	3.81 (0.653)	3.76 (0.023)	4.08 (0.692)	2.50 (0.500)
	2–3 per week	3.50 (1.632)	3.20 (1.367)	2.87 (1.389)	2.50 (1.193)
	Once per week	3.45 (1.564)	2.62 (1.213)	2.87 (1.342)	2.61 (1.297)
	2–3 per month	3.95 (0.757)	2.71 (0.894)	3.13 (0.912)	2.79 (1.130)
	One a month	4.04 (0.432)	2.64 (0.723)	3.22 (0.666)	3.04 (0.939)
	More rarely	3.99 (0.542)	2.76 (0.802)	3.30 (0.714)	2.97 (1.162)
*p*-value		0.039 *	0.343	0.484	0.887

* *p* < 0.05; ** *p* < 0.01; *** *p* < 0.001 ()—standard deviation.

## Data Availability

The data presented in this study are available on request from the corresponding author.
